# Repurposing antitussive benproperine phosphate against pancreatic cancer depends on autophagy arrest

**DOI:** 10.1002/1878-0261.12854

**Published:** 2020-12-12

**Authors:** Huanyu Zhang, Zhe Zhang, Yonghao Huang, Siyuan Qin, Li Zhou, Ningna Weng, Jiayang Liu, Mei Yang, Xiaodian Zhang, Yanda Lu, Lin Ma, Shaojiang Zheng, Qifu Li

**Affiliations:** ^1^ Key Laboratory of Emergency and Trauma of Ministry of Education & Tumor Institute the First Affiliated Hospital Hainan Medical University Haikou China; ^2^ Department of Neurology, the First Affiliated Hospital Hainan Medical University Haikou China; ^3^ School of Basic Medicine and Life Sciences Hainan Medical University Haikou China; ^4^ Key Laboratory of Brain Science Research & Transformation in Tropical Environment of Hainan Province Haikou China; ^5^ State Key Laboratory of Biotherapy and Cancer Center, West China School of Basic Medical Sciences & Forensic Medicine, Collaborative Innovation Center for Biotherapy West China Hospital, Sichuan University Chengdu China

**Keywords:** autophagy arrest, benproperine phosphate, drug repurposing, pancreatic cancer, RAB11A

## Abstract

Pancreatic cancer (PC) is one of the most common human malignancies worldwide and remains a major clinical challenge. Here, we found that benproperine phosphate (BPP), a cough suppressant, showed a significant anticancer effect on PC both *in vitro* and *in vivo* via the induction of autophagy‐mediated cell death. Mechanistic studies revealed that BPP triggered AMPK/mTOR‐mediated autophagy initiation and disturbed Ras‐related protein Rab‐11A (RAB11A)‐mediated autophagosome–lysosome fusion, resulting in excessive accumulation of autophagosomes. Inhibition of autophagy or overexpression of RAB11A partially reversed BPP‐induced growth inhibition in PC cells, suggesting that BPP might induce lethal autophagy arrest in PC cells. In conclusion, our results identify BPP as a potent antitumor agent for PC via the induction of autophagy arrest, therefore providing a new potential therapeutic strategy for the treatment of PC.

## Graphical abstract

Here, we show that benproperine phosphate (BPP), a cough suppressant, triggers AMPK/mTOR‐mediated autophagy initiation and disturbs Ras‐related protein Rab‐11A (RAB11A)‐mediated autophagosome–lysosome fusion, resulting in excessive accumulation of autophagosomes in pancreatic cancer (PC) cells. Inhibition of autophagy or overexpression of RAB11A partially reverses BPP‐induced growth inhibition in PC cells, suggesting BPP as a potent anti‐PC agent via the induction of autophagy arrest.

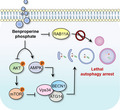

**Abbreviations**
3‐MA: 3‐methyladenine
AO: acridine orange
Baf A1: bafilomycin A1
BPP: benproperine phosphate
CQ: chloroquine
LDH: lactate dehydrogenase
PC: pancreatic cancer
RAB11A: Ras‐related protein Rab‐11A
RAPA: rapamycin

## 1 Introduction

Pancreatic cancer (PC) is one of the most intractable tumors with low survival rate and high mortality [1, 2, 3]. Currently, surgical resection is still generally recognized as the most effective clinical treatment of PC, which causes great suffering to patients regretfully, especially the elderly [4]. In addition, most PC patients are in late stage when diagnosed, in this case they are not suitable for resection [5]. Other therapeutic options, including chemotherapy and radiotherapy, have been shown considerable effects. However, overall prognosis of PC is largely unfavorable due to drug resistance or tumor recurrence that ultimately causes treatment failure [6, 7]. As such, there is an urgent need to develop novel therapeutic agents for efficient treatment of PC.

Autophagy is a multistep lysosomal degradation pathway in which dysfunctional cytoplasmic components are trafficked into autophagosomes and then degraded by autolysosomes depending on autophagosome–lysosome fusion [8, 9]. However, the role of autophagy in regulating the death or survival of cancer cells remains controversial [10]. As a prosurvival mechanism in most cases, autophagy facilitates tumor cell growth under chemotherapy‐induced stress, resulting in drug resistance [11]. Moreover, it has been widely documented that dysregulation of autophagy is closely linked to tumorigenesis [12]. Conversely, increasing evidence suggests that dysregulation of autophagy, such as lethal autophagy arrest, results in cell death and growth inhibition of tumor; thus, autophagy could be considered as a tumor‐suppression role [13]. Thus, understanding the paradoxical role of autophagy and the related mechanism involved in cancer is important for oncotherapy.

**Fig. 1:**
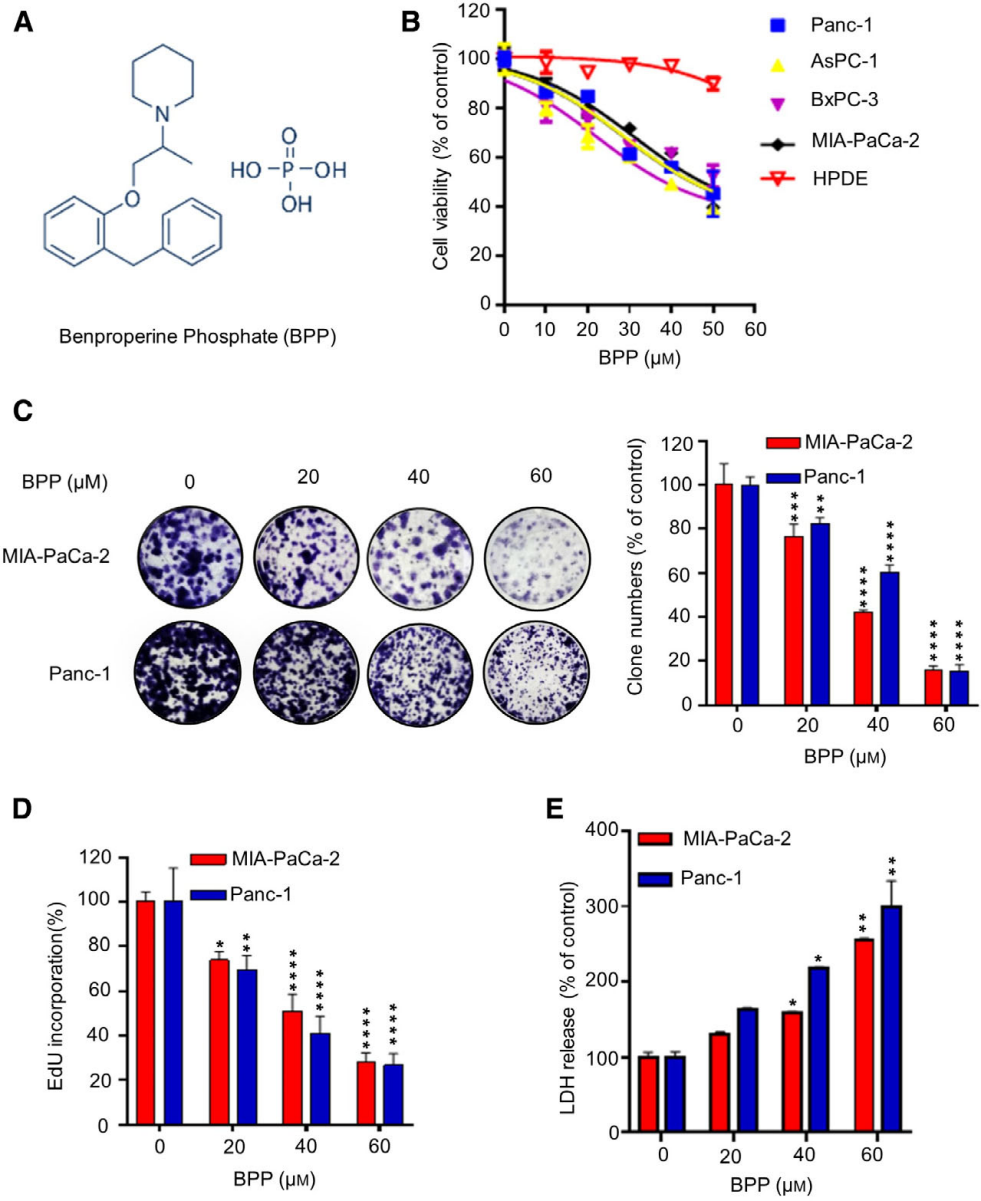
BPP inhibits PC cells growth *in vivo*. (A) Chemical structure of BPP. (B) Cell viability of various PC cell lines treated with indicated concentrations of BPP for 24 h. (C) Colony formation assay of PC cells treated with the indicated concentrations of BPP. Representative images (left) and quantification of colonies (right) were shown. (D‐E) EdU incorporation assay (D) and LDH release assay (E) of PC cells treated with the indicated concentrations of BPP for 24 h. Results are representative of three independent experiments. All data are shown as mean ± SD. The *P* values were determined by two‐tailed *t*‐test. *P < 0.05; **P < 0.01; ***P < 0.001; ****P < 0.0001.

Drug repurposing has recently drawn growing attention in the cancer management with favorable therapeutic effects [14, 15]. Several classes of antifungal and antiparasitic agents have shown great potential to be repurposed for anticancer application [16, 17]. Benproperine phosphate (BPP) (Fig. 1A), which is widely used as a nonproductive cough suppressant, has been reported to exhibit anticancer activities in several cancer cells [18, 19]. It has been reported that BPP inhibits cancer metastasis through regulation of actin‐related protein 2/3 complex subunit 2 (ARPC2)‐mediated pathway or inhibition of angiogenesis. In addition, BPP also shows suppressive effects on PC cells both *in vitro* and *in vivo* [19]. However, the underlying mechanisms remain poorly understood.

In this study, we demonstrate the anticancer effect of BPP for PC treatment both *in vitro* and *in vivo*. Interestingly, we find that BPP induces autophagy initiation and autophagosome formation via regulating AMPK/mTOR pathway. Meanwhile, BPP inhibits autophagosome–lysosome fusion by downregulating Ras‐related protein Rab‐11A (RAB11A) level leading to excessive accumulation of autophagosomes and lethal autophagy arrest. Our study reveals a novel anticancer mechanism for BPP and demonstrates the therapeutic potential of BPP for the treatment of PC.

## 2 Materials and methods

### 2.1 Cell culture

Human PC MIA‐PaCa‐2, Panc‐1, AsPC‐1, BxPC‐3 cells, and human normal pancreatic duct human pancreatic ductal epithelial cells (HPDE) cells were kindly provided by Stem Cell Bank, Chinese Academy of Science. Cell culture grows in a humidified atmosphere at 37 °C and 5% CO_2_. MIA‐PaCa‐2, Panc‐1 cell lines were cultured in high glucose Dulbecco's modified Eagle's medium (Gibco, Carlsbad, CA, USA); other cell lines were cultured in RPMI medium 1640. Both two culture mediums were supplemented with 10% FBS (Biowest, Loire Valley, France), 1% penicillin/streptomycin (Hyclone, Logan, UT, USA).

### 2.2 Reagents and antibodies

Reagents used in this study from the following resources: BPP (S5256) and Z‐VAD‐FMK (S7023) were purchased from Selleck (Houston, TX, USA). Chloroquine (CQ; HY‐17589), 3‐methyladenine (3‐MA; HY‐19312), bafilomycin A1 (Baf A1; HY‐100558), rapamycin (RAPA; HY‐10219) were purchased from MedChem Express (Monmouth Junction, NJ, USA). The 3‐(4, 5‐dimethylthiazol‐2‐yl)‐2, 5‐diphenyltetrazolium bromide (MTT) (M2128), DMSO (D2650), and Crystal Violet (C0775) were obtained from Millipore Sigma (Darmstadt, Germany). Baf A1, RAPA, and Z‐VAD‐FMK were dissolved in DMSO. BPP, 3‐MA, MTT, and CQ diphosphate salt were dissolved in PBS. The antibodies used in this study were as follows: anti‐ATG5 (12994S), anti‐Beclin 1 (3738), anti‐mTOR (2972), anti‐p‐mTOR (Ser2448) (2971), anti‐p70S6K (9202), anti‐p‐p70S6K (Ser371) (9208), anti‐4EBP1 (9452), anti‐p‐4EBP1 (Ser65) (9451), and anti‐RAB11A (2413S) were obtained from Cell Signaling Technology (Boston, MA, USA); anti‐LC3 (PM036; MBL (Nagoya, Aichi, Japan), anti‐Ki67 (SAB5600249; Millipore Sigma); anti‐P62 (sc‐48402), anti‐β‐actin (sc‐1616), horseradish peroxidase‐conjugated anti‐rabbit secondary antibody (sc‐2004) and horseradish peroxidase‐conjugated anti‐mouse secondary antibody (sc‐2005) were obtained from Santa Cruz Biotechnology (Dallas, TX, USA). For immunofluorescence, goat anti‐rabbit Alexa Fluor 488 and goat anti‐mouse Alexa Fluor 594 were obtained from Invitrogen (Carlsbad, CA, USA).

### 2.3 Detection of cell growth

BPP‐treated cell growth was measured using the MTT assay. Cells were plated in 96‐well plates (4 × 10^3^ cells/well) and different treatments for 24 h. The optical density of each cell culture well was measured by spectrophotometry at a wavelength of 570 nm. For the colony formation assay, cells were plated in 24‐well plates (1000 cells/well) with different treatments. After 1 week, cells were fixed with 4% paraformaldehyde in PBS for 1 h and washed three times by PBS, then stained with crystal violet for 30 min, and washed three times by PBS. The visible colonies were photographed with Molecular Imager Gel *Doc* XR + System (Bio‐Rad, CA, USA), and the number of colonies was counted using imagej software (NIH, Bethesda, MD, USA). The EdU incorporation assay kit (RiboBio Co., Ltd, C10310, Guangzhou, China) was used to detect cell proliferation, and the detail operation as described before [20].

### 2.4 Lactate dehydrogenase release assay

The cytotoxicity under different treatments was assessed by using the lactate dehydrogenase (LDH) release kit (C0016; Beyotime, Shanghai, China) as previously described. The studies were performed according to the instructions provided by the supplier.

### 2.5 Western blotting analysis

Cells lysates were prepared with RIPA lysis buffer (150 mm Tris/HCl, pH 7.5, 150 mm NaCl, 1% NP‐40, and protease inhibitors) and quantified by bicinchoninic acid Protein Assay (Thermo Fisher Scientific, 23250; Waltham, MA, USA). Equal amounts of proteins (15–30 μg) were used for immunoblotting assay. Proteins were separated on SDS/PAGE and transferred to poly(vinylidene difluoride) membranes (EMD Millipore, ISEQ00010), then blocking with skimmed milk in TBST (Thermo Fisher Scientific, 28360). After blocking, the membranes were incubated with primary antibodies at 4 °C overnight and then were incubated with the secondary antibodies for 1.5 h at room temperature. Enhanced Chemiluminescence reagents (EMD Millipore, WBKLS0500) were used to examine the target proteins.

### 2.6 Immunofluorescence

Cells were grown on glass coverslips in 24‐well plates (5 × 10^3^ cells/well). After different treatments, cells were fixed with 4% paraformaldehyde in PBS for 1 h and washed three times by PBS, and then, cells were incubated with 0.4% Triton X‐100 (Sigma‐Aldrich, 9002‐93‐1) for permeabilization. Cells were then incubated with 5% BSA (ST023; Beyotime) for 30 min to blocking nonspecific binding sites of primary antibody. After being incubating with primary antibodies at 4 °C overnight treated cells were incubated with the Alexa Flour secondary antibodies for 1 h at room temperature. Nucleus was dyed with DAPI (C0060; Solarbio; Beijing, China) for 10 min. Images were captured with a confocal laser scanning microscopy (Carl Zeiss, Oberkochen, Germany).

### 2.7 RNA interference

*ATG5*, *BECN1*, *Bcl‐2,* and scramble small interfering RNA (siRNA) were synthesized by GenePharma (Shanghai, China). The sequences of siRNA were as follows: human *ATG5* siRNA, 5′‐GCAACUCUGGAUGGGAU‐UGTT‐3′; human *BECN1* siRNA, 5′‐CAGUUUGGCACAAUCAAUAT‐3′; human *Bcl‐2* siRNA, 5′‐GUGAAGUCAACAUGCCUGCTT‐3′. siRNAs were transfected into cells by using Lipofectamine 3000 reagent (Thermo Fisher Scientific). The transfection time was 48 h and the detail operations described as the manufacturer's protocol.

### 2.8 Acridine orange staining

Cells were cultured in 24‐well plates (5 × 10^3^ cells/well). After different treatment, cells were then stained with acridine orange (AO; 1 mg·mL^−1^) (A6014; Sigma‐Aldrich) in PBS at 37 °C for 15 min. Cells were washed with PBS and then observed under fluorescence microscopy (Olympus Optical Co., Hamburg, Germany).

### 2.9 Lyso‐Tracker Red staining

Cells in different treatments groups were collected and then incubated with Lyso‐Tracker Red (C1046; Beyotime) at 37 °C for 30 min in the dark. After washing with PBS, at least 10^4^ live cells were analyzed on the FACS‐Calibur flow cytometer (Becton Dickinson, Franklin Lake, NJ, USA). Data analysis was performed with flowjo software (Becton Dickinson). Cells were also visualized by using the confocal laser scanning microscopy (Carl Zeiss).

### 2.10 Tumor xenograft model

Male BALB/c nude mice (HFK Bioscience, Beijing, China), 5 weeks old and 18–20 g each, were raised under specific pathogen free conditions. For the subcutaneous PC xenograft model, Panc‐1 cells (7 × 10^6^ cells/mouse) were suspended in PBS and injected subcutaneously into mice. When the tumor volume reached ~ 100 mm^3^, the mice were randomly divided into vehicle and treatment groups. BPP or physiologic saline was administered 5 days per week (50 mg·kg^−1^, oral gavage). Measuring the length (l) and width (w) of tumors every other day and calculating the volume (mm^3^) (V = l × w^2^/2). After 21 days of treatment, mice were euthanized. Tumor tissues were collected and then fixed in 4% paraformaldehyde immediately. All animal experiments were approved by the Institutional Animal Care and Treatment Committee of Sichuan University.

### 2.11 Immunohistochemistry

Immunohistochemical staining was performed as described previously [21]. To performing the quantitative scoring analysis, the percentage of staining‐positive cells area (A) was multiplied by the immunostaining intensity (B: 0, negative; 1, weakly positive; 2, positive; 3, strongly positive). The final score for each slide was calculated as A × B. All samples were visualized by using a DM2500 fluorescence microscope (Danaher, Wetzlar, Germany).

### 2.12 Statistical analysis

All statistical analysis and graphics were performed using graphpad 7 software (GraphPad, La Jolla, CA, USA). One‐way ANOVA or Student's *t*‐test was used to analyze statistical differences. All data are presented as the mean ± SD from at least three individual experiments. A value of *P* < 0.05 was considered as statistically significant.

## 3 Results

### 3.1 BPP inhibits the growth of PC cells *in vitro*

To determine whether BPP exhibits antitumor effect against PC, we examined cell growth in BPP‐treated PC cells. As shown in Fig. 1B, BPP significantly decreased the cell viability of PC cell lines in a dose‐dependent manner. In contrast, BPP showed no obvious cytotoxicity in HPDE. Consistently, colony formation (Fig. 1C) and EdU incorporation assay (Fig. 1D) revealed that BPP treatment inhibited the proliferation of PC cells. In addition, increased LDH release was observed by LDH release assay in BPP‐treated PC cells (Fig. 1E). Together, these results suggest that BPP has antitumor effect in PC cells *in vitro*.

### 3.2 BPP induces autophagy initiation in PC cells

We next explored the mechanism by which BPP inhibits PC cell growth. We used the inhibitors of cell death in different forms during BPP treatment [22, 23]. The results showed that CQ (a lysosomal inhibitor), rather than z‐VAD‐FMK (an apoptosis inhibitor) or ferrostatin‐1 (a ferroptosis inhibitor), had significant influence on PC cell growth in the presence of BPP (Fig. S1A,B). These data implied that autophagy may be involved in BPP‐mediated growth inhibition in PC cells.

**Fig. 2:**
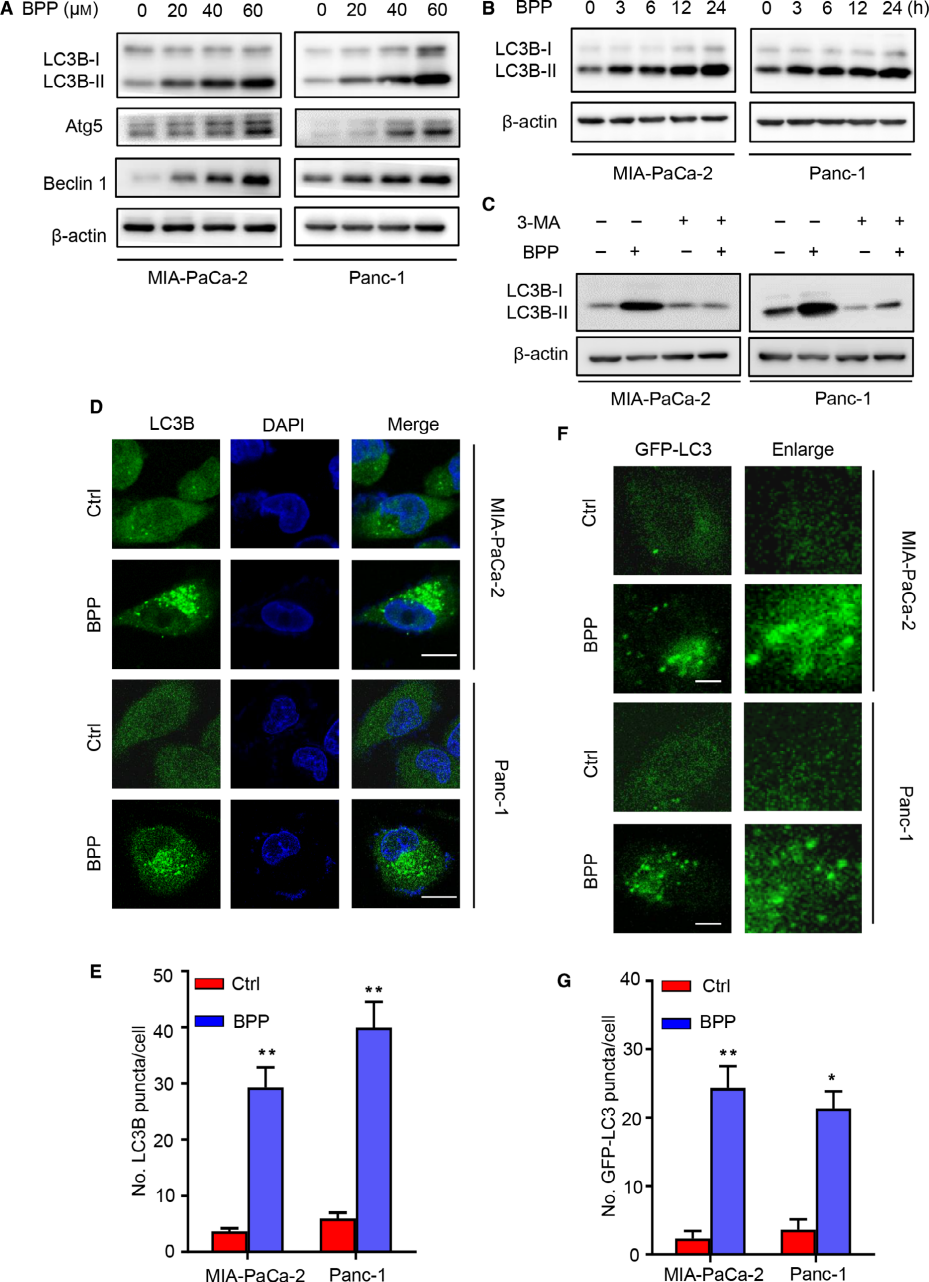
BPP induces autophagy initiation in PC cells. (A) Immunoblot analysis of LC3B‐I, LC3B‐II, Atg5, and Beclin 1 in PC cells treated with indicated concentrations of BPP for 24 h. (B) Immunoblotting analysis of LC3B‐II accumulation in PC cells treated with 40 μm BPP for indicated time. (C) Immunoblotting analysis of LC3B‐I and LC3B‐II in PC cells treated with or without 40 μm BPP in the presence or absence of 10 mm 3‐MA for 24 h. (D‐E) Immunofluorescence analysis (D) of LC3B in PC cells treated with or without 40 µM BPP for 24 h. Scale bars, 10 μm. The number of LC3B puncta (E) was shown. (F‐G) PC cells were transiently transfected with GFP‐tagged LC3 (GFP‐LC3) construct and then treated with 40 μm BPP for 24 h (F). Scale bars, 10 μm. The number of GFP‐LC3B puncta (G) was shown. Results are representative of three independent experiments. All data are shown as mean ± SD. The *P* values were determined by two‐tailed *t*‐test. *P < 0.05; **P < 0.01.

We further determined whether BPP induces autophagy in PC cells by examining LC3B‐II accumulation (a hallmark of autophagy) [24] and levels of autophagy‐related proteins. As shown in Fig. 2A, BPP indeed induced autophagy initiation, as evidenced by dose‐dependent increases in the levels of LC3B‐II, Atg5, and Beclin 1 in PC cells. Furthermore, the BPP‐induced LC3B‐II accumulation was time‐dependent (Fig. 2B). Treatment with 3‐MA (a class III PI3K inhibitor) (Fig. 2C), or knockdown of *BECN1* (Fig. S2A) or *ATG5* (Fig. S2B) inhibited LC3B‐II accumulation in BPP‐treated PC cells. Consistently, a significant accumulation of endogenous LC3B puncta was observed in BPP‐treated cells by LC3B immunofluorescence staining (Fig. 2D,E). To confirm this observation, GFP‐tagged LC3B plasmid was used, and, a marked increase of GFP‐LC3B puncta was observed in BPP‐treated cells compared with control cells (Fig. 2F,G). In addition, AMPK/mTOR pathway was activated in BPP‐treated PC cells (Fig. S2C). Together, these results indicate that BBP induces autophagy initiation in PC cells.

### 3.3 BPP blocks autophagic flux in PC cells

**Fig. 3:**
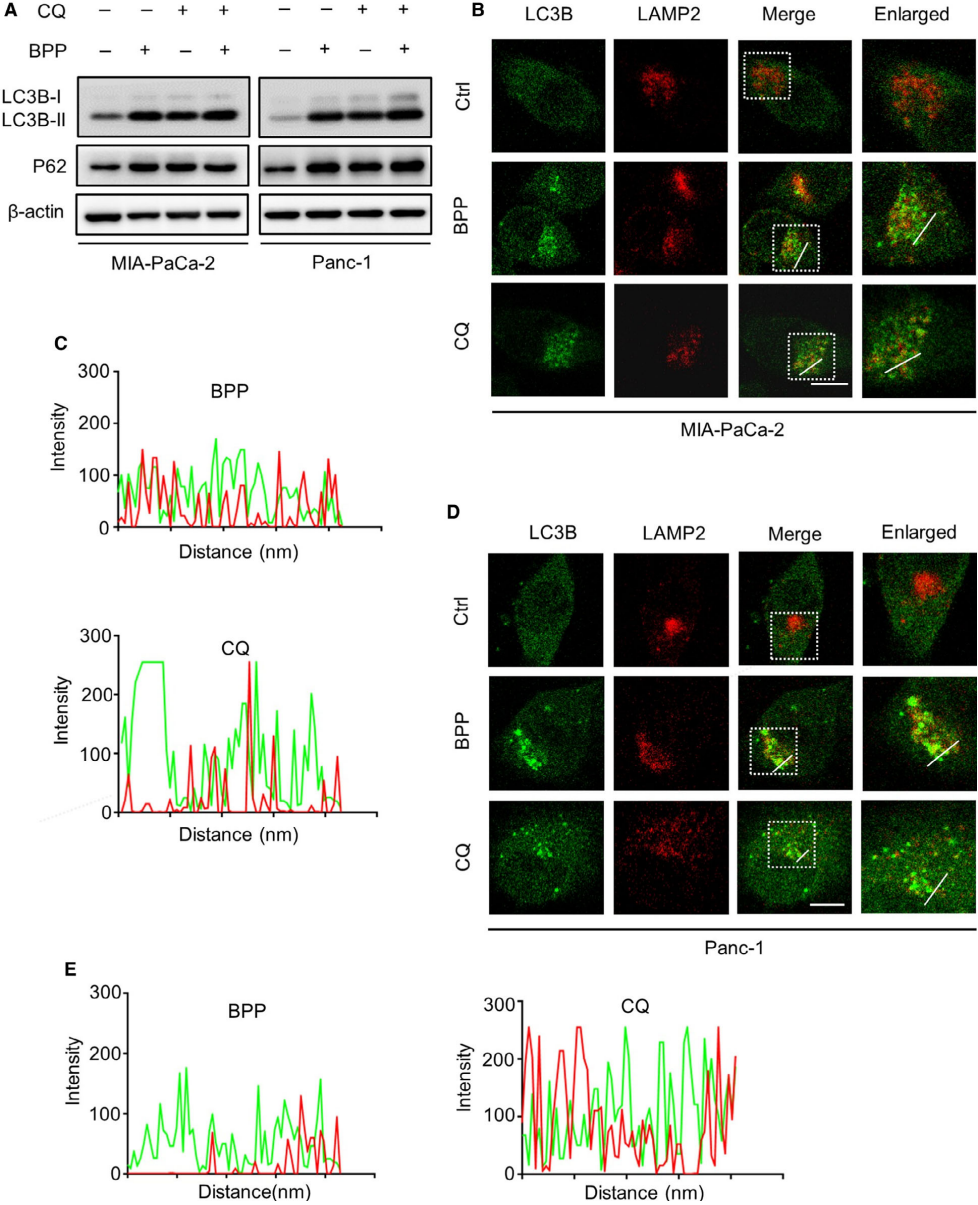
BPP blocks autophagic flux in PC cells. (A) Immunoblotting analysis of LC3B and P62 expression in PC cells treated with or without 40 μm BPP in the presence or absence of 10 μm CQ for 24 h. (B) Immunofluorescent analysis of the colocalization of endogenous LC3B with LAMP2 after treatment of 40 μm BPP or 10 μm CQ for 24 h in MIA‐PaCa‐2 cells. Scale bars, 10 μm. (C) The fluorescence intensity corresponding to LC3B and LAMP2 was shown. (D) Immunofluorescent analysis of the colocalization of endogenous LC3B with LAMP2 after treatment of 40 μm BPP or 10 μm CQ for 24 h in Panc‐1 cells. Scale bars, 10 μm. (E) The fluorescence intensity corresponding to LC3B and LAMP2 was shown. Results are representative of three independent experiments.

The accumulation of autophagosomes in cells may result from increased autophagy initiation or impaired autophagic flux, or both. To determine whether BPP induced complete autophagic flux, we investigated the protein expression of LC3B‐II and P62 (a classical substrate of autophagy degradation) by using CQ in BPP‐treated cells. The data showed that BPP treatment elevated the protein levels of LC3B‐II and P62, whereas CQ treatment could not further increase the levels of LC3B‐II and P62 in BPP‐treated PC cells (Fig. 3A). Moreover, the colocalization of LC3B with LAMP2 (a lysosome marker) was not observed in BPP‐treated PC cells (Fig. 3B‐E), suggesting no fusion of the autophagosome with lysosome. Moreover, using a tandem mRFP‐GFP‐tagged LC3 construct, we found that BPP significantly induced the accumulation of GFP^+^RFP^+^ signal, implying increased autophagosomes (Fig. S3A‐D). Furthermore, using leupeptin (Leup), a protease inhibitor, in the presence or absence of BPP in PC cells, we found that BPP treatment markedly inhibited GFP^‐^RFP^+^ signal induced by Leup (Fig. S3A‐D). Taken together, these findings indicate that BPP inhibits autophagosome–lysosome fusion in PC cells, resulting in impaired autophagic flux.

### 3.4 BPP inhibits autophagosome–lysosome fusion by downregulating RAB11A in PC cells

**Fig. 4:**
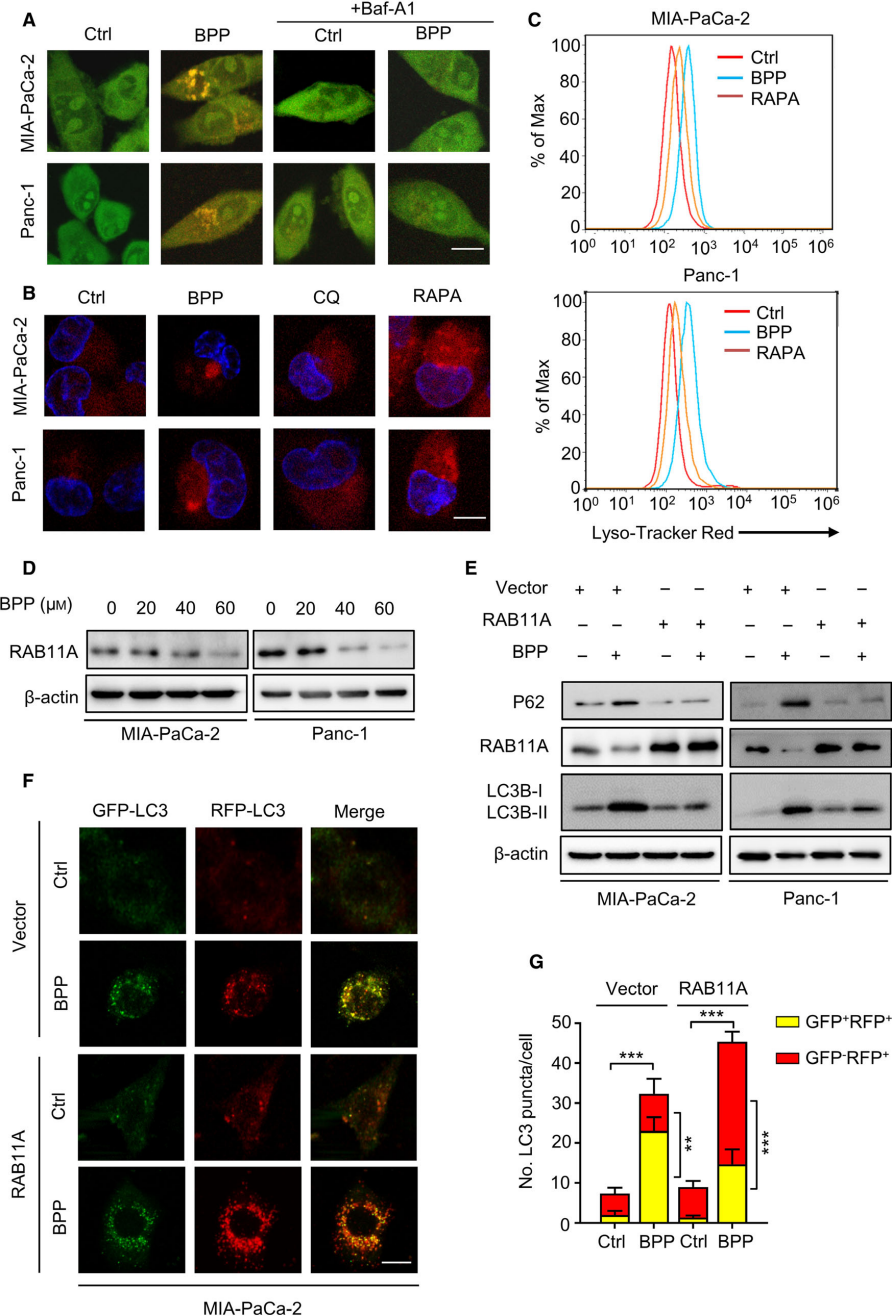
BPP blocks autophagic flux by downregulating RAB11A in PC cells. (A) PC cells treated with or without 40 μm BPP in the presence or absence of 100 nm Baf‐A1 for 24 h, then stained with 1 μm AO for 15 min. (B) PC cells treated with 40 μm BPP, 10 μm CQ, or 100 nm RAPA for 24 h, then stained with 75 nm Lyso‐Tracker Red for 30 min. (C) FACS analysis of Lyso‐Tracker Red after PC cells were treated with 40 μm BPP or 100 nm RAPA for 24 h. (D) Immunoblot analysis of RAB11A expression in PC cells treated with indicated concentrations of BPP for 24 h. (E) Immunoblotting LC3B‐I, LC3B‐II, P62, and RAB11A expression in PC cells transfected with empty vector or RAB11A plasmid for 48 h, followed by treatment with or without 40 μm BPP for another 24 h. (F‐G) MIA‐PaCa‐2 cells were transfected with mRFP‐GFP‐LC3 and empty vector or RAB11A plasmid for 48 h, followed by treatment with or without 40 μm BPP for another 24 h (F). Scale bars, 10 μm. The number of autophagosomes (GFP^+^RFP^+^) and autolysosomes (GFP^‐^RFP^+^) per cell (G) was quantified. Results are representative of three independent experiments. All data are shown as mean ± SD. The *P* values were determined by two‐tailed *t*‐test. **P < 0.01; ***P < 0.001.

As the pH of the acidic compartments is a key factor of autophagosome–lysosome fusion [25], we thus examined the number of acidic lysosomes by staining with AO (a dye of intracellular acidic vesicles). BPP treatment induced a significant increase of acid vesicles, while combinatorial treatment of BPP with Baf‐A1 (a potent selective inhibitor of V‐ATPase) resulted in the reduction of acidic vesicles (Fig. 4A). Consistently, BPP treatment induced an obvious increase of the number of acidic lysosomes as evidenced by Lyso‐Tracker Red staining (Fig. 4B,C). Thus, our data indicate that lysosomal acidification is not the driving force for BPP‐mediated inhibition of autophagosome–lysosome fusion.

Rab GTPases, such as RAB11A, have been reported to play key roles in the autophagosome–lysosome fusion [26, 27]. We thus examined RAB11A expression following BPP treatment in PC cells and found a decreased expression of RAB11A in a dose‐dependent manner (Fig. 4D). Enforced expression of RAB11A partially compromised the increased levels of P62 induced by BPP (Fig. 4E), suggesting that downregulation of RAB11A may be involved in BPP‐induced impairment of autophagic flux. These results were further strengthened by increased colocalization of LC3B with LAMP2 in response to BPP treatment in RAB11A‐overexpressing PC cells (Fig. S4A‐D). Consistently, using a tandem mRFP‐GFP‐tagged LC3 construct, we found that BPP‐induced accumulation of autophagosomes (GFP^+^RFP^+^ signal) was decreased by RAB11A overexpress (Figs 4F,G and S4E,F). Taken together, these results suggest that BPP impairs autophagosome–lysosome fusion by downregulating RAB11A.

### 3.5 BPP induces lethal autophagosome accumulation in PC cells

**Fig. 5:**
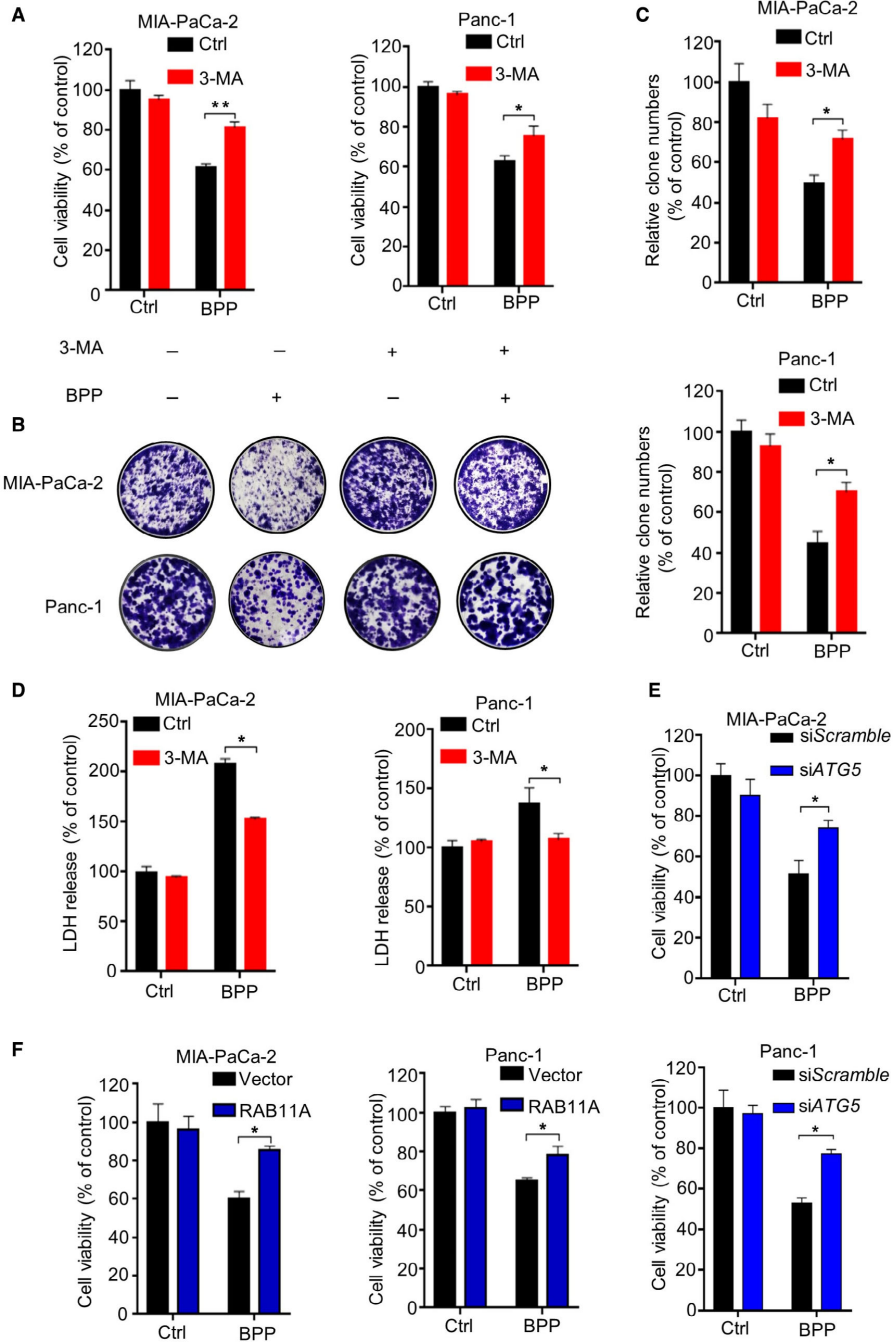
BPP induces lethal autophagosome accumulation in PC cells. (A) Cell viability of PC cells treated with or without 40 μm BPP in the presence or absence of 10 mm 3‐MA for 24 h. (B‐C) Colony formation assay of PC cells treated with or without 40 μm BPP in the presence or absence of 10 mm 3‐MA for 24 h (B). Quantification of colonies (C) was shown. (D) LDH release assay of PC cells treated with or without 40 μm BPP in the presence or absence of 10 mm 3‐MA for 24 h. (E) Cell viability of PC cells transfected with si*ATG5* or si*Scramble* followed by treatment with or without 40 μm BPP for 24 h. (F) Cell viability of PC cells transfected empty vector or RAB11A plasmid for 48 h, followed by treatment with or without 40 μm BPP for another 24 h. Results are representative of three independent experiments. All data are shown as mean ± SD. The *P* values were determined by two‐tailed *t*‐test. *P < 0.05; **P < 0.01.

To investigate the role of autophagy in PC cell growth upon BPP treatment, we treated PC cells with BPP in combination with 3‐MA. We found that 3‐MA treatment markedly restored BPP‐repressed growth of PC cells, as evidenced by MTT (Fig. 5A) and colony formation assay (Fig. 5B,C). In addition, combinatorial treatment of 3‐MA markedly decreased BPP‐induced cytotoxicity as evidenced by LDH release assay (Fig. 5D). Consistently, knockdown of *ATG5* or *BECN1* compromised BPP‐induced growth inhibition (Figs 5E and S5A). Of note, RAB11A overexpression partially abrogated growth inhibition in BPP‐treated PC cells (Fig. 5F). Furthermore, combinatorial treatment of BPP with si*Bcl‐2* had no obvious effect on BPP‐induced tumor suppression, indicating that Bcl‐2 was not involved in the anticancer effect of BPP in PC cells (Fig. S5B). Overall, these findings indicate that BPP induces autophagic cell death in PC cells.

### 3.6 BPP exhibits antitumor effect against PC cells *in vivo*

**Fig. 6:**
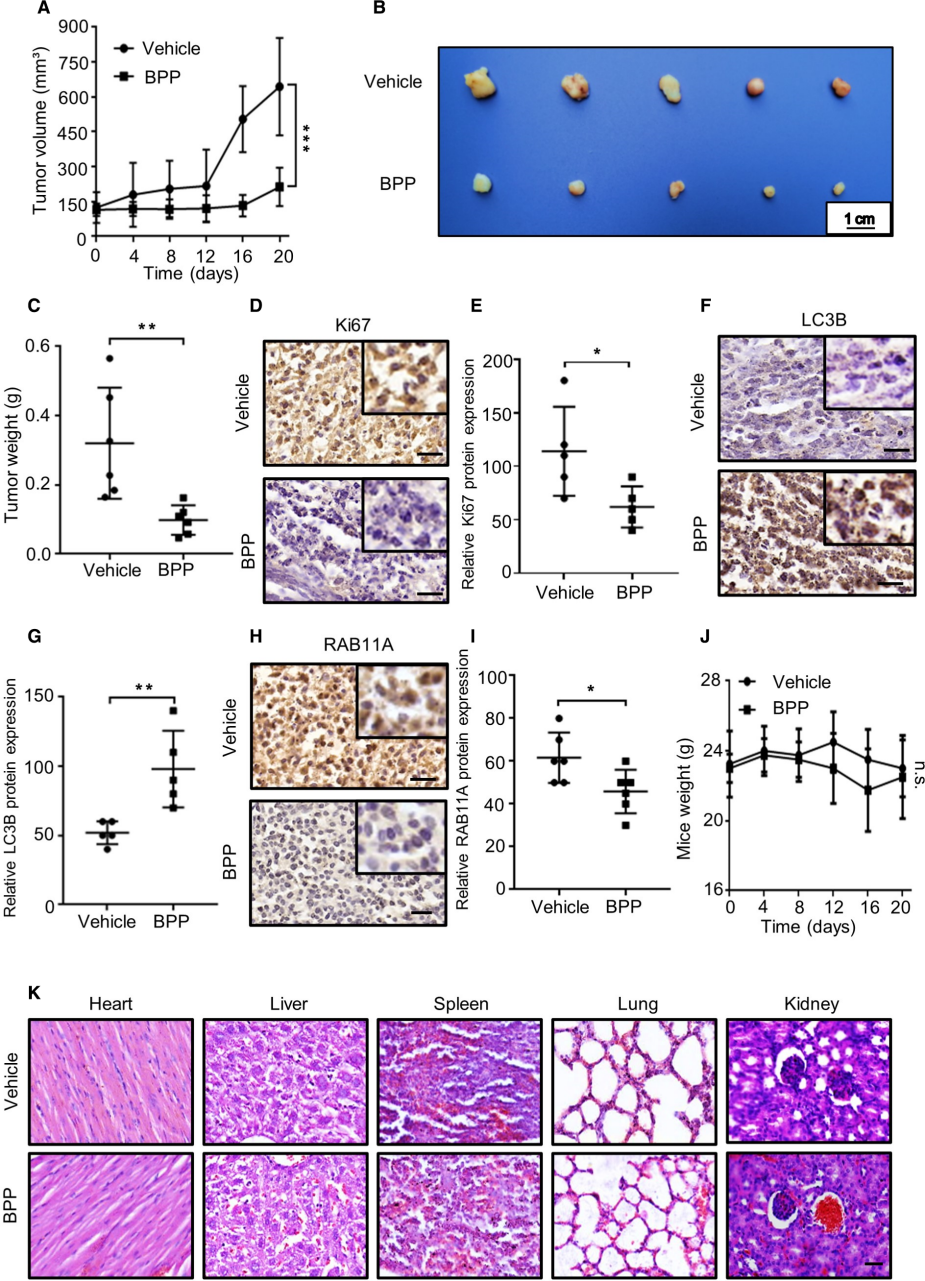
BPP exhibits antitumor effect against PC cells *in vivo*. (A‐C) 7 × 10^6^ Panc‐1 cells were injected subcutaneously into male nude mice. When the tumor volumes reached around 100 mm^2^, mice were received vehicle or BPP (five mice per group). The tumor volume was measured at the indicated time points (A). The image (B) and (C) weight of the tumor were shown. (D‐I) Immunohistochemistry staining and immunohistochemical scores of Ki67 (D‐E), LC3B (F‐G), and RAB11A (H‐I) were shown. Scale bar, 50 μm. (J) The body weight of mice treated with vehicle or BPP was measured at the indicated time points. (K) Hematoxylin and eosin staining of major organs in mice treated with vehicle or BPP. Scale bars, 50 μm. Results are representative of three independent experiments. All data are shown as mean ± SD. The *P* values were determined by two‐tailed *t*‐test. *P < 0.05; **P < 0.01; ***P < 0.001; ns, no statistical significance.

To further confirm the anticancer effect of BPP *in vivo*, a xenograft model was generated by subcutaneously inoculating the human Panc‐1 cells into nude mice. As expected, BPP treatment markedly reduced the growth rate (Fig. 6A), size (Fig. 6B), and weight (Fig. 6C) of PC xenografts. Consistently, weaker Ki67 immunohistochemical staining was observed in BPP‐treated mice compared to the vehicle‐treated group (Fig. 6D,E). Otherwise, we found that the expression of LC3B was increased (Fig. 6F,G) and the expression of RAB11A (Fig. 6H,I) was decreased in xenografts from BPP‐treated mice by immunohistochemical staining. Of note, BPP treatment did not significantly influence the mice body weight (Fig. 6J). Moreover, H&E staining of major organs showed no obvious toxic effect in BPP‐treated mice (Fig. 6K). Taken together, these results demonstrate that BPP inhibits the growth of PC cells *in vivo* and has no obvious toxicity in mice.

## 4 Discussion

Benproperine phosphate is a nonproductive cough suppressant with no obvious side effect [28]. Recently, BPP has been reported to exhibit anticancer effects in a variety of tumor models [19]. In this study, we demonstrated that BPP significantly suppressed cell proliferation in PC both *in vitro* and *in vivo*. We also showed that BPP markedly induced autophagy initiation via regulating AMPK/mTOR pathway and subsequently somehow increased the protein levels of Atg5 and Beclin 1 (maybe through enhancing their transcriptional levels or inhibiting their degradation), contributing to autophagy initiation. Combinatorial treatment of autophagic inhibitor 3‐MA or knockdown of autophagy‐related protein by siRNA decreased BPP‐mediated growth inhibition. We further revealed that BPP perturbed the fusion of autophagosomes with lysosomes by reducing the expression of RAB11A, thus resulting in an excessive accumulation of autophagosomes and cell death. Our study implies BPP as a potential candidate for PC treatment.

Drug repurposing is defined as a strategy of using existing drugs to redeveloping new therapies [15, 29]. With unexpected therapeutic effects in cancer treatment, drug repurposing has received increasing attention in recent years [14]. An earlier representative repurposed agent is CQ, an antimalarial drug, which has been used as an anticancer drug through regulation of autophagy [30]. Recently, accumulating data have shown that metformin (the first‐line treatment option for type 2 diabetes) and aspirin (a reliever or antipyretic) exhibit anticancer effects via novel molecular mechanism in many tumors [31, 32]. Our previous work has repurposed several classes of antifungal and antiparasitic agents as the potential candidates for anticancer application [16, 33]. In this study, we found that BPP inhibited the growth of PC by inducing autophagy arrest, suggesting that BPP was a potential candidate for drug repurposing.

Generally, autophagy arrest is due to the blockade of autophagic flux in cancer cells, which results in excessive accumulation of autophagosomes and cell death [8]. It has been reported that elaiophylin blocks autophagic flux and promotes the accumulation of autophagosome by attenuating the lysosomal cathepsin activity, resulting in cell death [34]. Our previous studies have also reported that regorafenib induces lethal autophagy arrest in glioblastoma [13]. In concordance with these results, our findings indicated that autophagy arrest partially contributed to BPP‐induced growth inhibition of PC cells, suggesting that targeting autophagy is a potential therapeutic strategy for the PC therapy.

The lysosomal acidity is necessary for intact autophagic flux in cancer cells. The dysregulation of lysosomal acidity results from the inactivation of related hydrolases, subsequently leading to the blockage of autophagic flux [25, 26]. Herein, we preferentially excluded the influence of lysosomal acidification in BPP‐induced autophagy arrest. Of note, a series of Rab GTPases which regulate autophagosome formation, such as Rab11A, are also involved in the blockage of autophagic flux in response to cellular stresses [35]. It has been reported that RAB11A is overexpressed in cancers and promotes cancer progression. The dysregulation of RAB11A partially contributes to activated autophagic flux, which accelerates the tumorigenesis and development in various tumors [13, 36]. Notably, our previous study identifies RAB11A as a potent regulator for autophagy arrest in response to regorafenib treatment [13]. Consistently, our findings indicated that BPP blocked autophagic flux by downregulating of RAB11A. Enforced expression of RAB11A could partially reverse the blockage of autophagic flux and alleviated cell death in BPP‐treated PC cells. These findings, that the dual effect of BPP functions as an inducer of autophagy initiation and inhibitor of autophagosome–lysosome fusion, also explained contradictory results caused by inhibiting different stages of autophagy. However, whether activated autophagic flux is related to overexpression of RAB11A, or RAB11A is a classical target in autophagy‐dependent PC, the detailed molecular mechanism needs a further verification.

## 5 Conclusion

In this study, we demonstrate that BPP inhibits the growth of PC cells both *in vitro* and *in vivo*. BPP induces autophagy initiation by the regulation of AMPK/mTOR pathways. Interestingly, BPP disturbs the fusion of autophagosomes with lysosomes by downregulating the expression of RAB11A, thus leading to excessive accumulation of autophagosomes and cell death. Our study suggests BPP as a potential repurposed nononcology drug for PC treatment.

## Conflict of interest

The authors declare no conflict of interest.

## Author contributions

QL, SZ, and ZZ conceived and designed the study; HZ, SQ, and NW acquired the data; HZ, ZZ, YH, JL, and MY analyzed and interpreted the data; HZ, ZZ, YH, and LZ drafted the article; HZ, ZZ, YH, LZ, YL, LM, and YC reviewed and/or revised the manuscript; QL and SZ supervised the study.

### Peer Review

The peer review history for this article is available at https://publons.com/publon/10.1002/1878‐0261.12854.

## Supporting information


**Fig. S1.** BPP induces PC cell death through induction of autophagy.
**Fig. S2.** BPP induces autophagy initiation in PC cells.
**Fig. S3.** BPP blocks autophagic flux in PC cells.
**Fig. S4.** Overexpression of RAB11A partially recovered the autophagosome‐lysosome fusion blockage in BPP‐treated PC cells.
**Fig. S5.** Inhibition of autophagy compromised BPP‐induced cell death in PC cells.

## References

[1] Chen W , Zheng R , Baade PD , Zhang S , Zeng H , Bray F , Jemal A , Yu XQ & He J (2016) Cancer statistics in China, 2015. CA Cancer J Clin 66, 115–132. 26808342 10.3322/caac.21338

[2] Ryan DP , Hong TS & Bardeesy N (2014) Pancreatic adenocarcinoma. N Engl J Med 371, 1039–1049. 25207767 10.1056/NEJMra1404198

[3] Siegel RL , Miller KD & Jemal A (2019) Cancer statistics, 2019. CA Cancer J Clin 69, 7–34. 30620402 10.3322/caac.21551

[4] Hidalgo M (2010) Pancreatic cancer. N Engl J Med 362, 1605–1617. 20427809 10.1056/NEJMra0901557

[5] Kleeff J & Michl P (2017) Targeted therapy of pancreatic cancer: biomarkers are needed. Lancet Oncol 18, 421–422. 28259609 10.1016/S1470-2045(17)30087-6

[6] Conroy T , Hammel P , Hebbar M , Ben Abdelghani M , Wei AC , Raoul J‐L , Choné L , Francois E , Artru P , Biagi JJ *et al* (2018) FOLFIRINOX or gemcitabine as adjuvant therapy for pancreatic cancer. N Engl J Med 379, 2395–2406. 30575490 10.1056/NEJMoa1809775

[7] Hezel AF , Kimmelman AC , Stanger BZ , Bardeesy N & Depinho RA (2006) Genetics and biology of pancreatic ductal adenocarcinoma. Genes Dev 20, 1218–1249. 16702400 10.1101/gad.1415606

[8] Galluzzi L & Green DR (2019) Autophagy‐independent functions of the autophagy machinery. Cell 177, 1682–1699. 31199916 10.1016/j.cell.2019.05.026 PMC7173070

[9] Levine B & Kroemer G (2019) Biological functions of autophagy genes: a disease perspective. Cell 176, 11–42. 30633901 10.1016/j.cell.2018.09.048 PMC6347410

[10] Singh SS , Vats S , Chia AY , Tan TZ , Deng S , Ong MS , Arfuso F , Yap CT , Goh BC , Sethi G *et al* (2018) Dual role of autophagy in hallmarks of cancer. Oncogene 37, 1142–1158. 29255248 10.1038/s41388-017-0046-6

[11] Gomez VE , Giovannetti E & Peters GJ (2015) Unraveling the complexity of autophagy: potential therapeutic applications in Pancreatic Ductal Adenocarcinoma. Semin Cancer Biol 35, 11–19. 26408419 10.1016/j.semcancer.2015.09.011

[12] Yang A , Herter‐Sprie G , Zhang H , Lin EY , Biancur D , Wang X , Deng J , Hai J , Yang S , Wong KK *et al* (2018) Autophagy sustains pancreatic cancer growth through both cell‐autonomous and nonautonomous mechanisms. Cancer Discov 8, 276–287. 29317452 10.1158/2159-8290.CD-17-0952 PMC5835190

[13] Jiang J , Zhang L , Chen H , Lei Y , Zhang T , Wang Y , Jin P , Lan J , Zhou L , Huang Z *et al* (2020) Regorafenib induces lethal autophagy arrest by stabilizing PSAT1 in glioblastoma. Autophagy 16, 106–122. 30909789 10.1080/15548627.2019.1598752 PMC6984601

[14] Pushpakom S , Iorio F , Eyers PA , Escott KJ , Hopper S , Wells A , Doig A , Guilliams T , Latimer J , McNamee C *et al* (2019) Drug repurposing: progress, challenges and recommendations. Nat Rev Drug Discov 18, 41–58. 30310233 10.1038/nrd.2018.168

[15] Zhang Z , Zhou L , Xie N , Nice EC , Zhang T , Cui Y & Huang C (2020) Overcoming cancer therapeutic bottleneck by drug repurposing. Signal Transduct Target Ther 5, 113. 32616710 10.1038/s41392-020-00213-8 PMC7331117

[16] Chen Y , Chen HN , Wang K , Zhang L , Huang Z , Liu J , Zhang Z , Luo M , Lei Y , Peng Y *et al* (2019) Ketoconazole exacerbates mitophagy to induce apoptosis by downregulating cyclooxygenase‐2 in hepatocellular carcinoma. J Hepatol 70, 66–77. 30287340 10.1016/j.jhep.2018.09.022

[17] Dou Q , Chen HN , Wang K , Yuan K , Lei Y , Li K , Lan J , Chen Y , Huang Z , Xie N *et al* (2016) Ivermectin induces cytostatic autophagy by blocking the PAK1/Akt axis in breast cancer. Cancer Res 76, 4457–4469. 27302166 10.1158/0008-5472.CAN-15-2887

[18] Li Y , Zhong DF , Chen SW & Maeba I (2005) Identification of some benproperine metabolites in humans and investigation of their antitussive effect. Acta Pharmacol Sin 26, 1519–1526. 16297353 10.1111/j.1745-7254.2005.00212.x

[19] Yoon YJ , Han YM , Choi J , Lee YJ , Yun J , Lee SK , Lee CW , Kang JS , Chi SW , Moon JH *et al* (2019) Benproperine, an ARPC2 inhibitor, suppresses cancer cell migration and tumor metastasis. Biochem Pharmacol 163, 46–59. 30710516 10.1016/j.bcp.2019.01.017

[20] Zhou L , Gao W , Wang K , Huang Z , Zhang L , Zhang Z , Zhou J , Nice EC & Huang C (2019) Brefeldin A inhibits colorectal cancer growth by triggering Bip/Akt‐regulated autophagy. FASEB J 33, 5520–5534. 30668917 10.1096/fj.201801983R

[21] Zhang Z , Gao W , Zhou L , Chen Y , Qin S , Zhang L , Liu J , He Y , Lei Y , Chen HN *et al* (2019) Repurposing brigatinib for the treatment of colorectal cancer based on inhibition of ER‐phagy. Theranostics 9, 4878–4892. 31410188 10.7150/thno.36254 PMC6691391

[22] Fink SL & Cookson BT (2005) Apoptosis, pyroptosis, and necrosis: mechanistic description of dead and dying eukaryotic cells. Infect Immun 73, 1907–1916. 15784530 10.1128/IAI.73.4.1907-1916.2005 PMC1087413

[23] Wang S , Wang H & Ding WX (2018) Pyroptosis, a novel player for alcoholic hepatitis? Hepatology 67, 1660–1662. 29222919 10.1002/hep.29725 PMC5906175

[24] Green DR & Levine B (2014) To be or not to be? How selective autophagy and cell death govern cell fate. Cell 157, 65–75. 24679527 10.1016/j.cell.2014.02.049 PMC4020175

[25] Kawai A , Uchiyama H , Takano S , Nakamura N & Ohkuma S (2007) Autophagosome‐lysosome fusion depends on the pH in acidic compartments in CHO cells. Autophagy 3, 154–157. 17204842 10.4161/auto.3634

[26] Lorincz P & Juhasz G (2019) Autophagosome‐lysosome fusion. J Mol Biol 432, 2462–2482. 31682838 10.1016/j.jmb.2019.10.028

[27] Murrow L & Debnath J (2015) ATG12‐ATG3 connects basal autophagy and late endosome function. Autophagy 11, 961–962. 25998418 10.1080/15548627.2015.1040976 PMC4502820

[28] Feng S , Liu J , Han X & Fan J (2009) Resonance light scattering study on the interaction of benproperine phosphate with eriochrome blue black R in the presence of sodium dodecylbenzene sulphonate and its analytical application. Luminescence 24, 67–72. 18800357 10.1002/bio.1064

[29] Nowak‐Sliwinska P , Scapozza L & Altaba ARI (2019) Drug repurposing in oncology: compounds, pathways, phenotypes and computational approaches for colorectal cancer. Biochim Biophys Acta Rev Cancer 1871, 434–454. 31034926 10.1016/j.bbcan.2019.04.005 PMC6528778

[30] Al‐Bari MA (2015) Chloroquine analogues in drug discovery: new directions of uses, mechanisms of actions and toxic manifestations from malaria to multifarious diseases. J Antimicrob Chemother 70, 1608–1621. 25693996 10.1093/jac/dkv018 PMC7537707

[31] Hua H , Zhang H , Kong Q , Wang J & Jiang Y (2019) Complex roles of the old drug aspirin in cancer chemoprevention and therapy. Med Res Rev 39, 114–145. 29855050 10.1002/med.21514

[32] Flory J & Lipska K (2019) Metformin in 2019. JAMA 321, 1926–1927. 31009043 10.1001/jama.2019.3805 PMC7552083

[33] Liu R , Li J , Zhang T , Zou L , Chen Y , Wang K , Lei Y , Yuan K , Li Y , Lan J *et al* (2014) Itraconazole suppresses the growth of glioblastoma through induction of autophagy: involvement of abnormal cholesterol trafficking. Autophagy 10, 1241–1255. 24905460 10.4161/auto.28912 PMC4203550

[34] Zhao X , Fang Y , Yang Y , Qin Y , Wu P , Wang T , Lai H , Meng L , Wang D , Zheng Z *et al* (2015) Elaiophylin, a novel autophagy inhibitor, exerts antitumor activity as a single agent in ovarian cancer cells. Autophagy 11, 1849–1863. 25893854 10.1080/15548627.2015.1017185 PMC4824600

[35] Ao X , Zou L & Wu Y (2014) Regulation of autophagy by the Rab GTPase network. Cell Death Differ 21, 348–358. 24440914 10.1038/cdd.2013.187 PMC3921601

[36] Liao Y , Li M , Chen X , Jiang Y & Yin X‐M (2018) Interaction of TBC1D9B with mammalian ATG8 homologues regulates autophagic flux. Sci Rep 8, 13496. 30202024 10.1038/s41598-018-32003-2 PMC6131546

