# Supplementary material for: Repurposing antitussive benproperine phosphate against pancreatic cancer depends on autophagy arrest
Source: Mol Oncol. 2020 Dec 12;15(2):725–38. doi: 10.1002/1878-0261.12854 (PMC7858282; doi:10.1002/1878-0261.12854)
Supplement: Supplementary file 1 — Fig. S1. BPP induces PC cell death through induction of autophagy. Fig. S2. BPP induces autophagy initiation in PC cells. Fig. S3. BPP blocks autophagic flux in PC cells. Fig. S4. Overexpression of RAB11A partially recovered the autophagosome‐lysosome fusion blockage in BPP‐treated PC cells. Fig. S5. Inhibition of autophagy compromised BPP‐induced cell death in PC cells. [file MOL2-15-725-s001.docx]

**Supplementary Material**

**Repurposing antitussive benproperine phosphate against pancreatic cancer depends on autophagy arrest**

Huanyu Zhang^1,2,3,4*^, Zhe Zhang^5*^, Yonghao Huang^1*^, Siyuan Qin^5^, Li Zhou^5^, Ningna Weng^5^, Jiayang Liu^5^, Mei Yang^5^, Xiaodian Zhang^1^, Yanda Lu^1^, Lin Ma^2,4^, Shaojiang Zheng^1#^, Qifu Li^1,2,3,4#^

1. *Key Laboratory of Emergency and Trauma of Ministry of Education & Tumor Institute of the First Affiliated Hospital of Hainan Medical University, Haikou 571199, P.R. China*

2*. Department of Neurology, the First Affiliated Hospital, Haikou 570102, P. R. China*

3. *School of Basic Medicine and Life Sciences, Hainan Medical University, Haikou 571199, P. R. China*

4*. Key Laboratory of Brain Science Research & Transformation in Tropical Environment of Hainan Province, Haikou 571199, P.R. China.*

5*. State Key Laboratory of Biotherapy and Cancer Center, West China Hospital, and West China School of Basic Medical Sciences & Forensic Medicine, Sichuan University, and Collaborative Innovation Center for Biotherapy, Chengdu, 610041, P. R. China*

*Equal contribution.
^#^Address correspondence to:

Qifu Li: The First Affiliated Hospital of Hainan Medical University, Haikou, P. R. China. Tel: 0086898-66786797. Fax: 0086898-66772248. E-mail: [lee-chief@163.com](mailto:lee-chief@163.com) Shaojiang Zheng: Key Laboratory of Emergency and Trauma of Ministry of Education & Tumor Institute of the First Affiliated Hospital of Hainan Medical University, 3 Xueyuan Road, Longhua District, Haikou, 571199, P.R. China. Tel: 86-0898-66892302. E-mail: zhengsj2008@163.com

**Running Title:** Benproperine phosphate inhibits pancreatic cancer

**Disclosure of Potential Conflicts of Interest:** No potential conflicts of interest were disclosed.


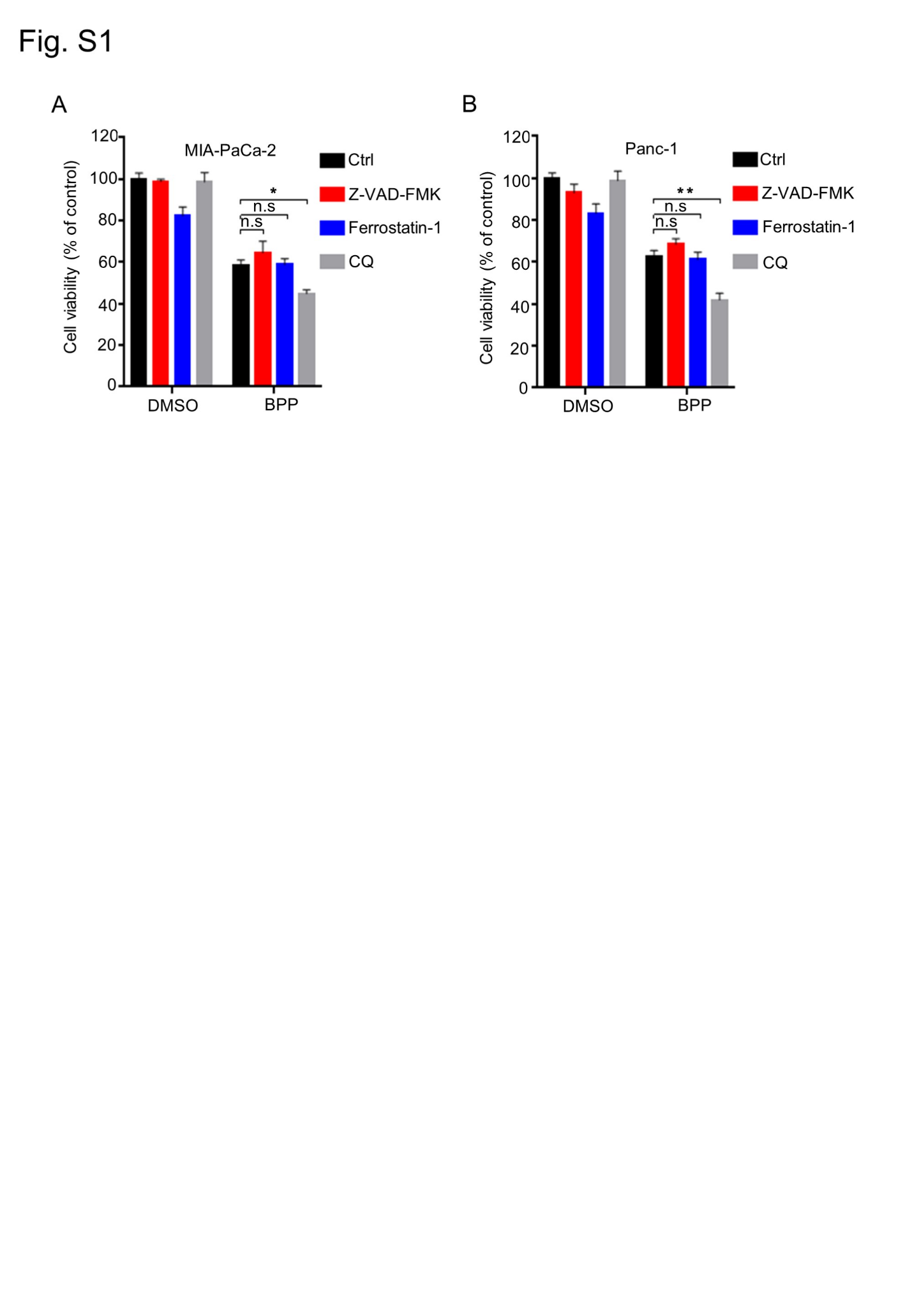


**Fig. S1 BPP induces PC cell death through induction of autophagy.** **(A-B)** Cell viability of MIA-PaCa-2 (A) and Panc-1 (B) cells treated with 40 μM BPP in combination with or without the different inhibitors (ferrostatin-1, 0.4 μM, Z-VAD-FMK, 40 μM, CQ, 10 μM). Results are representative of three independent experiments. All data are shown as mean ± SD. The *P* values were determined by two-tailed t-test. *, *P* < 0.05; **, *P* < 0.01; ns, no statistical significance.


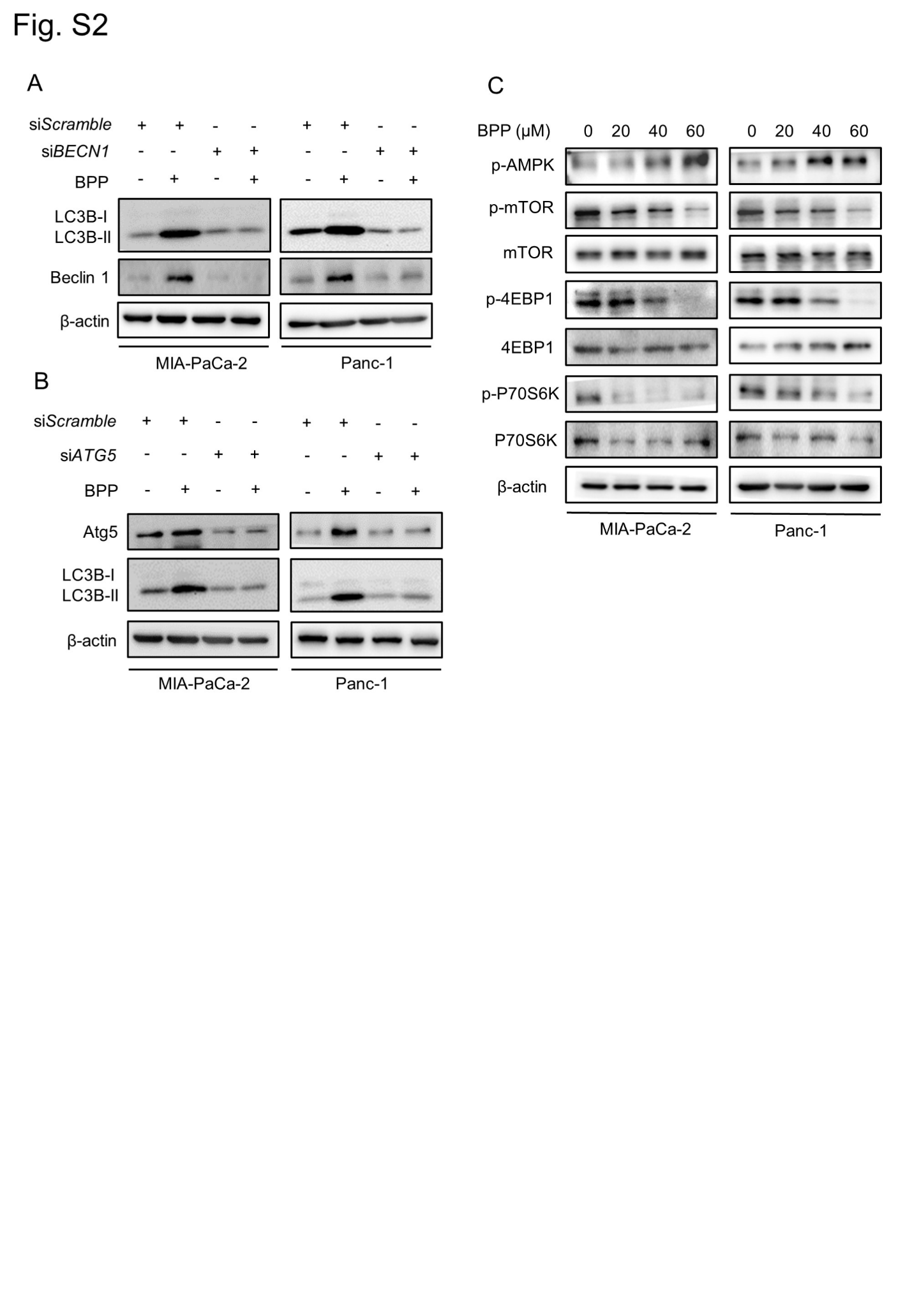


**Fig. S2 BPP induces autophagy initiation in PC cells.** **(A)** Immunoblotting of LC3B and Beclin 1 in PC cells transfected with si*BECN1* or si*Scramble* followed by treatment with or without 40 μM BPP for 24 h. **(B)** Immunoblotting of LC3B and Atg5 in PC cells transfected with si*ATG5* or si*Scramble* followed by treatment with or without 40 μM BPP for 24 h. **(C)** Immunoblot analysis of p-AMPK, p-mTOR, mTOR, p-4EBP1, 4EBP1, p-P70S6K, P70S6K in PC cells treated with indicated concentrations of BPP for 24 h. Results are representative of three independent experiments.


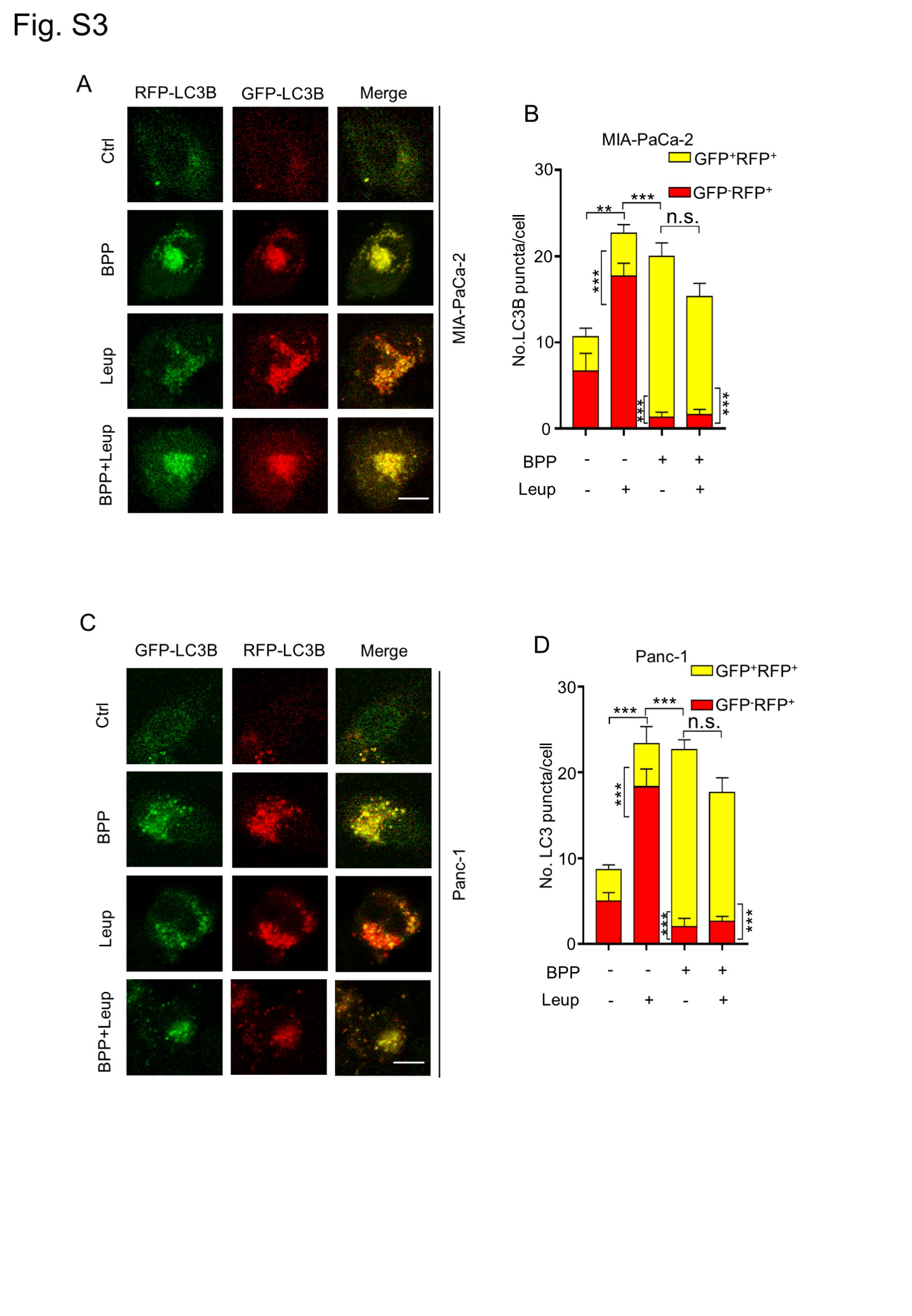


**Fig. S3 BPP blocks autophagic flux in PC cells. (A-B)** MIA-PaCa-2 cells were transfected with mRFP-GFP-LC3 for 48 h, and treated with 40 μM BPP for 24 h in the presence or absence of 10 μM Leup for 12 h (A). Scale bars, 10 μm. The number of autophagosomes (GFP^+^RFP^+^) and autolysosomes (GFP^-^RFP^+^) per cell (B) was quantified. Results are representative of three independent experiments. All data are shown as mean ± SEM. The *P* values were determined by two-tailed t-test. **, *P*<0.01; ***, *P*<0.001; ns, no statistical significance. **(C-D)** Panc-1 cells were transfected with mRFP-GFP-LC3 for 48 h, and treated with 40 μM BPP for 24 h in the presence or absence of 10 μM Leup for 12 h (B). Scale bars, 10 μm. The number of autophagosomes (GFP^+^RFP^+^) and autolysosomes (GFP^-^RFP^+^) per cell (C) was quantified. Results are representative of three independent experiments. All data are shown as mean ± SD. The *P* values were determined by two-tailed t-test. **, *P*<0.01; ***, *P*<0.001; ns, no statistical significance.


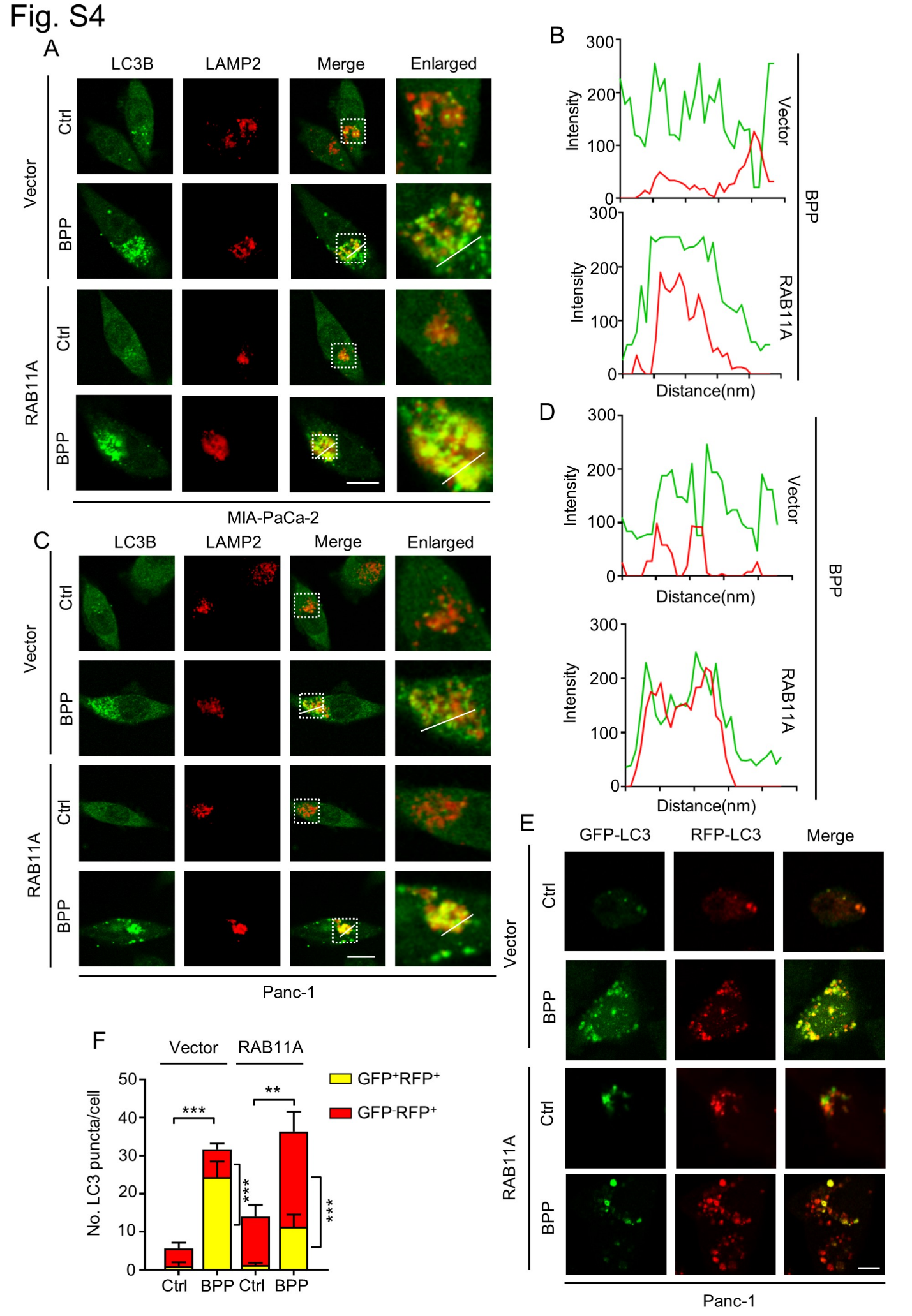


**Fig. S4 Overexpression of RAB11A partially recovered the autophagosome-lysosome fusion blockage in BPP-treated PC cells.** **(A)** Immunofluorescent analysis of the colocalization of endogenous LC3 and LAMP2 in MIA-PaCa-2 cells transfected empty vector or RAB11A plasmid for 48 h, followed by treatment with or without 40 μM BPP for another 24 h. Scale bars, 10 μm. **(B)** The fluorescence intensity corresponding to LC3B and LAMP2 was shown. **(C)** Immunofluorescent analysis of the colocalization of endogenous LC3 and LAMP2 in Panc-1 cells transfected empty vector or RAB11A plasmid for 48 h, followed by treatment with or without 40 μM BPP for another 24 h. Scale bars, 10 μm. **(D)** The fluorescence intensity corresponding to LC3B and LAMP2 was shown. **(E-F)** Panc-1 cells were transfected with mRFP-GFP-LC3 and empty vector or RAB11A plasmid for 48 h, followed by treatment with or without 40 μM BPP for another 24 h (E). Scale bars, 10 μm. The number of autophagosomes (GFP^+^RFP^+^) and autolysosomes (GFP^-^RFP^+^) per cell (F) was quantified. Results are representative of three independent experiments. All data are shown as mean ± SD. The *P* values were determined by two-tailed t-test. **, *P*<0.01; ***, *P*<0.001.


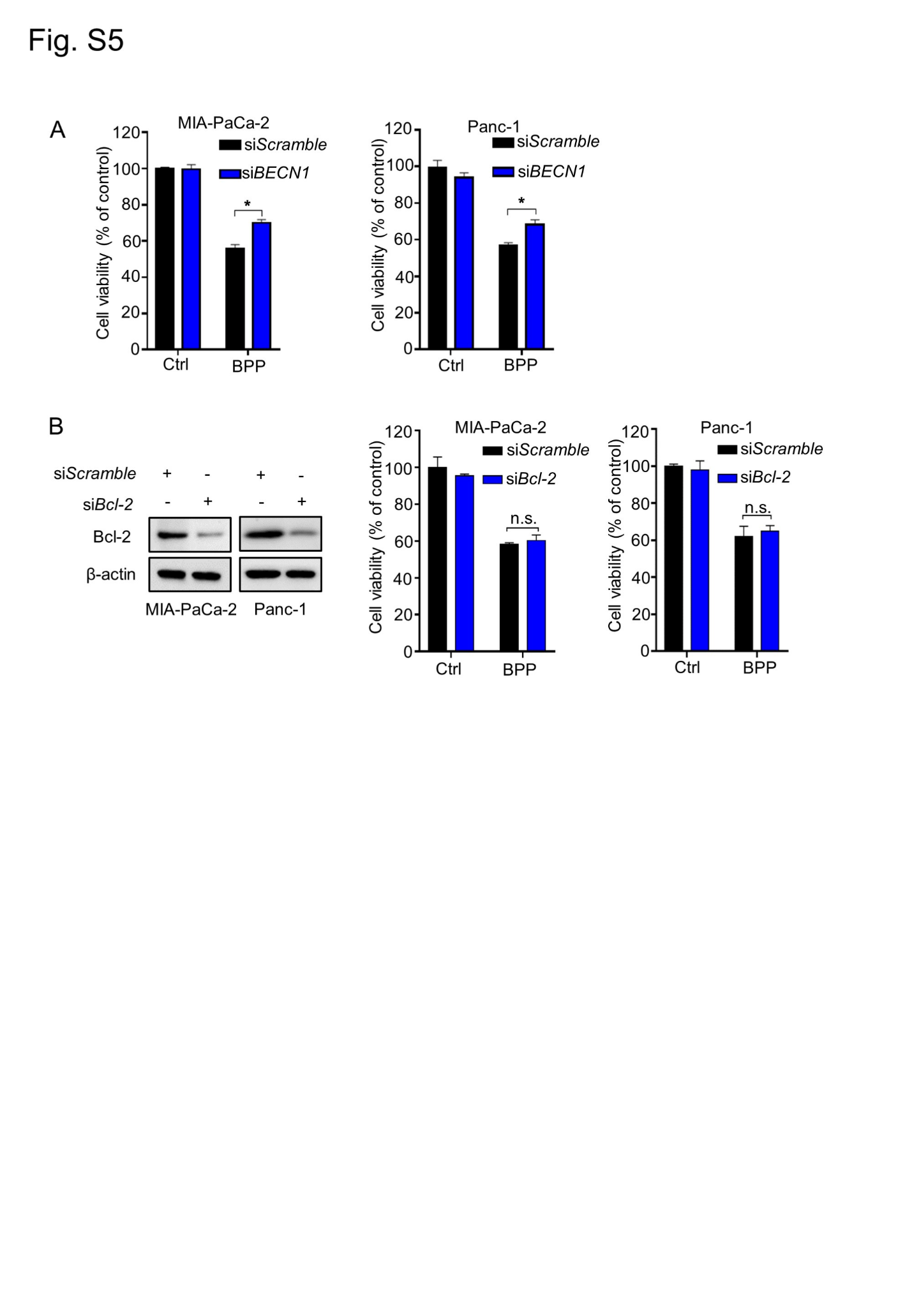


**Fig. S5 Inhibition of autophagy compromised BPP-induced cell death in PC cells. (A)** Cell viability of PC cells transfected with si*BECN1* or si*Scramble* followed by treatment with or without 40 μM BPP for 24 h. *, *P*<0.05. **(B)** Cell viability of PC cells transfected with si*Bcl-2* or si*Scramble* followed by treatment with or without 40 μM BPP for 24 h. Results are representative of three independent experiments. All data are shown as mean ± SD. The P values were determined by two-tailed t-test. ns, no statistical significance.
